# An Assessment of Surface Water Detection Methods for Water Resource Management in the Nigerien Sahel

**DOI:** 10.3390/s20020431

**Published:** 2020-01-12

**Authors:** Kelsey Herndon, Rebekke Muench, Emil Cherrington, Robert Griffin

**Affiliations:** 1 NASA SERVIR Science Coordination Office, NASA Marshall Space Flight Center, Huntsville, AL 35899, USA; 2 Earth System Science Center, The University of Alabama in Huntsville, Huntsville, AL 35899, USA; 3 Department of Atmospheric and Earth Science, The University of Alabama in Huntsville, Huntsville, AL 35899, USA

**Keywords:** remote sensing, spectral indices, Landsat 8 OLI, West Africa

## Abstract

Water is a scarce, but essential resource in the Sahel. Rainfed ephemeral ponds and lakes that dot the landscape are necessary to the livelihoods of smallholder farmers and pastoralists who rely on these resources to irrigate crops and hydrate cattle. The remote location and dispersed nature of these water bodies limits typical methods of monitoring, such as with gauges; fortunately, remote sensing offers a quick and cost-effective means of regularly measuring surface water extent in these isolated regions. Dozens of operational methods exist to use remote sensing to identify waterbodies, however, their performance when identifying surface water in the semi-arid Sahel has not been well-documented and the limitations of these methods for the region are not well understood. Here, we evaluate two global dynamic surface water datasets, fifteen spectral indices developed to classify surface water extent, and three simple decision tree methods created specifically to identify surface water in semi-arid environments. We find that the existing global surface water datasets effectively minimize false positives, but greatly underestimate the presence and extent of smaller, more turbid water bodies that are essential to local livelihoods, an important limitation in their use for monitoring water availability. Three of fifteen spectral indices exhibited both high accuracy and threshold stability when evaluated over different areas and seasons. The three simple decision tree methods had mixed performance, with only one having an overall accuracy that compared to the best performing spectral indices. We find that while global surface water datasets may be appropriate for analysis at the global scale, other methods calibrated to the local environment may provide improved performance for more localized water monitoring needs.

## 1. Introduction

In the Sahel, a semi-arid region spanning across northern sub-Saharan Africa, pastoralists and smallholder farmers rely heavily on rain-fed ponds and lakes for hydrating their cattle and irrigating small fields [1,2]. Many surface water bodies in the region are ephemeral and heavily dependent on precipitation, reaching capacity during the rainy season, which spans from May to October, and disappearing completely during the dry season [1,2,3]. In response to severe droughts in the 1970s and 1980s, the Nigerien government has implemented laws, regulations, and policies that dictate the interactions of pastoralists and smallholder farmers with surface water resources [4]. To better inform these policies, there is a need for more effective surface water monitoring; however, many of these features are remote and dispersed, often located in areas with low population densities, making them difficult to monitor via conventional methods, such as gauges or with total data stations. Remote sensing provides a means of monitoring these scattered waterbodies across time and over vast areas.

There are many operational methods used to identify inland surface water using remotely sensed data. In addition to pre-calculated global surface water datasets, algorithms for detecting surface water from remote sensing data also have been created and applied at global, regional, and local scales. These include the thresholding of single bands and spectral indices [5,6,7,8,9,10,11,12]; simple decision trees relying on knowledge of the spectral properties of water compared to other land cover types [13,14,15]; spectral mixture analysis (SMA) [16,17,18], and supervised and unsupervised classification schemes (including machine learning algorithms) [19,20]. 

The most common and simple method of identifying surface water using remotely sensed data is calculation of spectral indices. Many indices have been developed specifically to exploit the unique spectral signature of water compared to other land cover types [5,6,7,8,9,10,12,13]. While each index has strengths and weaknesses, it is not apparent which works best in a semi-arid environment, such as the Nigerien Sahel. Additionally, many of these methods require identifying an optimal threshold, which may vary across space and time due to relative land cover, atmospheric effects, and water quality [10]. Some methods have been specifically developed for threshold stability [3] or to avoid thresholding at all [21], however they have not been programmatically assessed for the Sahel. 

Previous studies are limited in their comparison of surface water detection methods, typically comparing relatively few indices, or simply presenting new methods without comparing them to existing ones (see [10,12,21,22,23,24,25] for examples). These comparisons offer little agreement over which spectral index performs the best. Relatively few of these comparison studies address surface water identification in the Sahel, which has unique land cover and water body characteristics considerations, e.g., high seasonal variability in the extent of surface water, large seasonal changes in relative land cover (including vegetation), and a great variety of water body types, ranging from clear to extremely turbid. One recent exception is Campos and colleagues’ [23] comparison of spectral index performance in Mauritania, which encompasses the Sahara-Sahel transition zone. While their study only looked at the capabilities of three spectral indices to determine the seasonality of surface water (permanent, non-water, and seasonal), the authors did note the poor performance of the Normalized Difference Water Index (NDWI) compared to the Normalized Difference Moisture Index (NDMI) and the Modified Normalized Difference Water Index (MNDWI) [23]. A more comprehensive comparison of potential methods along with their strengths and weaknesses, is necessary to guide water resource managers in using remote sensing to monitor these important features in the Sahel. 

In addition to spectral indices, preexisting global surface water datasets are also used to assess the distribution of surface water. These datasets leverage the global availability of many sources of remotely sensed data, such as Landsat and MODIS, to calculate surface water extent across the globe. These datasets can be static, representing a single moment in time or a summary statistic over a long period of time, or dynamic, providing time series information on surface water extent [26,27]. However, global datasets are often insufficient for mapping the small ephemeral water bodies scattered across the Nigerien Sahel, either because the dataset consists of static maps that do not capture the extreme seasonal dynamism of water bodies in the region or, if dynamic, they fail to detect the large number of small, turbid, spectrally complex ephemeral water bodies that many pastoralists and smallholder farmers regularly rely on and which might not make it into algorithm training datasets. While those omitted water bodies may not be significant in the context of wider, global-scale hydrologic processes, they are essential to local lifeways. 

This paper evaluates two commonly used 30-m-resolution global surface water datasets: the Joint Research Centre’s Global Surface Water (JRC GSW) [26] dataset and the Landsat QA water mask dataset [27]. Additionally, fifteen spectral indices developed for detecting surface water from remotely sensed data are calibrated and evaluated to determine which is best suited for identifying water bodies across space and time in the Sahel. Simple decision tree methods of surface water detection that have been developed specifically for water detection in a semi-arid environment are also applied and the strengths and weaknesses of each are discussed. 

## 2. Materials and Methods

### 2.1. Study Area

**Figure 1:**
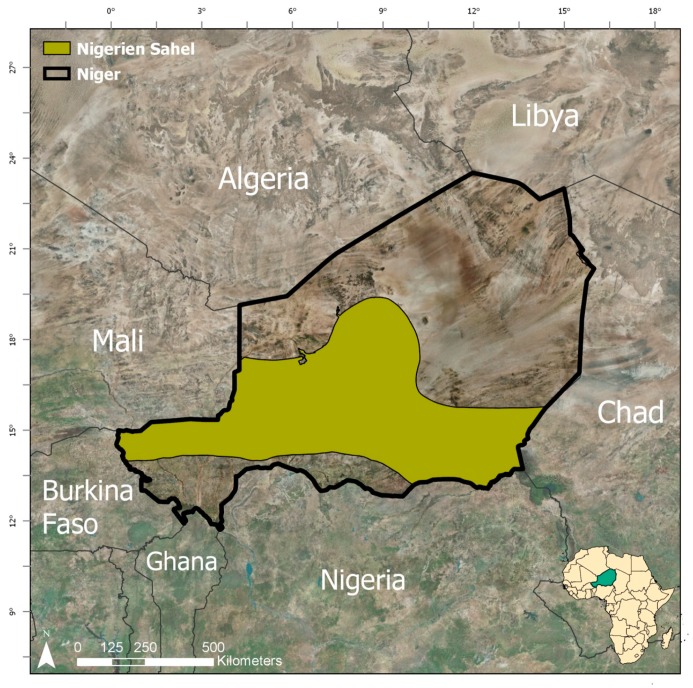
The Nigerien Sahel.

**Figure 2:**
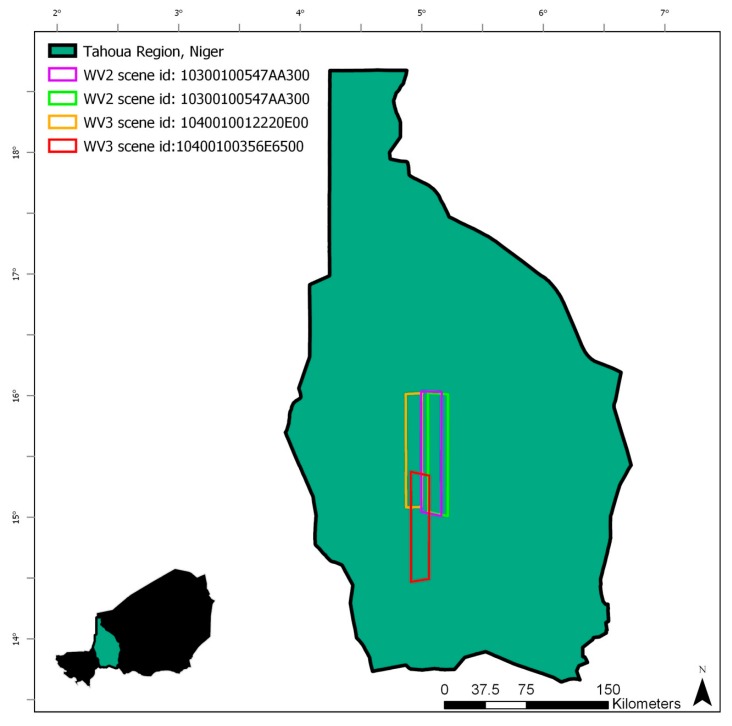
Study area (Tahoua Region) and DigitalGlobe WorldView imagery coverage.

Niger is a large landlocked country in west Africa, bordered by Mali and Burkina Faso to the west, Algeria and Libya to the north, Chad to the east, and Nigeria and Benin to the south (Figure 1). The study area is the Tahoua Region of Niger (Figure 2), which is located in the southwest of Niger and is crosscut by the Sahel, a large ecoregion that spans across Africa and is characterized by a semi-arid climate with seasonal vegetation and water bodies that are driven by a mono-modal pattern of annual precipitation that typically peaks in August. Annual precipitation in the Tahoua Region varies from north to south, with areas in the north receiving as little as 200 mm per year and areas in the southern portion of the Sahel receiving as much as 600 mm per year [28]. This pattern of precipitation is one of the main drivers of the filling and drying up of surface water bodies in the region. Vegetation also varies on this north-to-south axis, with small scale cultivation in the south and dry steppe in the north. The Tahoua Region on Niger is an important area for migrating pastoralists [1,2,4] who rely on dispersed water bodies for hydration throughout their journey.

### 2.2. Data

The recently released JRC GSW dataset [26] is one of the first global surface water datasets to attempt to capture the dynamism of surface water extent at a monthly time scale using high resolution (30 m) Landsat data. This dataset consists of various global surface water characteristics at 30 m resolution, including occurrence, occurrence change intensity, seasonality, recurrence, transitions, and maximum water extent. Additionally, monthly surface water extent maps, from which all of the other products are derived, can be found in the Google Earth Engine (GEE) [29] version of the JRC GSW database. The JRC GSW monthly surface water extent data, used for this study, were derived from the Landsat 5–8 series of satellites, spanning March 1984 to October 2015. The dataset was calculated on the GEE platform using a complex decision tree, including “expert systems, visual analytics, and evidential reasoning” [26]. For this study, only the October 2015 monthly surface water extent map was used to compare to reference data derived from the very high-resolution October 2015 Digital Globe (DG) data. 

The Landsat surface reflectance product produced by the United States Geological Survey (USGS) contains a quality control band which provides some land cover information, such as water, ice, and snow. This information is used in deriving the surface reflectance product using the Landsat Surface Reflectance Code (LaSRC), based on the Second Simulation of a Satellite Signal in the Solar Spectrum (6S) radiative transfer model. The pixel_qa band, generated by the CFMask algorithm, was used to generate surface water extent because it “is likely to present more accurate results than the internal tests LaSRC uses for cloud, cloud shadow, snow/ice, and water” [30,31]. The pixel_qa band was reclassified so that water pixels (values of 324, 388, 836, 900, and 1348) were reclassified with a value of 1; all other pixels were assigned a value of 0. 

Landsat 8 surface reflectance data derived from the LaSRC were used to calculate the surface water extent from the spectral indices and simple decision trees. This method of atmospheric correction was used because in preliminary studies it had little impact on the accuracy of surface water masks derived from spectral indices in the Sahel [32]. These data were procured through GEE from the United States Geological Society’s (USGS) LaSRC, which uses the 6S radiative transfer model [30,31]. 

High-resolution top-of-atmosphere reflectance imagery from the DG WorldView-2 and WorldView-3 satellites were used as reference data to assess the accuracy of the existing global surface water datasets to represent surface water in the Nigerien Sahel and for calibration and validation of the water masks derived from spectral indices. Table 1 presents all the specific images and dates used in this analysis. 

### 2.3. Methods

Creating the reference dataset: Calibration and validation of the surface water extraction methods were based on four very high-resolution images, totaling 6951.43 km^2^, from DigitalGlobe’s WorldView-2 and WorldView-3 satellites (Table 2). Due to the highly dynamic nature of ephemeral water bodies within the study area, DG imagery used for calibration and validation was only selected from dates corresponding to the exact dates of the Landsat imagery used. The coverage of the reference dataset was limited by the availability of DG data that overlapped spatially and temporally with Landsat imagery. 

The 2.5 m resolution WorldView-2 and 3 imagery was pansharpened to 0.5 m resolution using the Brovey transform in ArcGIS Pro. A 600 m by 600 m grid was placed over each DG scene to systematically identify surface water across the larger image. Each grid block was closely inspected and water bodies were visually identified in the DG true color image and hand-digitized in ArcGIS Pro to serve as the reference surface water extent. The resulting water body outline shapefiles were then converted to a 30 m resolution raster whereby each 30 m pixel that was covered by more than 50% water was classified as a water pixel and those with less than 50% water coverage were classified as a non-water pixel. Two sets of random points were generated for each DG image, stratified across water and non-water areas identified in the DG imagery, with each point being a minimum of 30 m apart. The number of sample points varied slightly (max 1300 points, min 1297 points) from scene to scene because of these constraints. The status of these points as water or non-water was compared with the values for the same points extracted from the water masks derived via the various surface water identification algorithms. Visual inspection revealed that this random selection process for water points yielded points representing both near-shore and mid-water body locations and for non-water points included sand, exposed bedrock, and vegetated areas. 

Evaluating global surface water datasets: Two existing global surface water datasets were assessed for their utility in identifying ephemeral water bodies in the Nigerien Sahel, the JRC GSW monthly surface water extent dataset [26] and the Landsat 8 QA FMask water layer [27]. The JRC GSW dataset was selected for October 2015 to coincide with the DG high resolution imagery from October 21, 2015. Data for the JRC GSW dataset are stored as 0, 1, or 2, with 2 being water, 1 being non-water, and 0 being no data. Four scenes of the Landsat 8 QA FMask water layer were compared to the corresponding reference dataset. For each global dataset, values for the sample points were compared through OA and producer and consumer accuracies. 

Calculating spectral indices and simple decision trees: Fifteen spectral indices were calculated for each of the four Landsat 8 OLI surface reflectance scenes. Calculations were automated in ArcGIS Pro. These indices were selected because they are either traditionally used to monitor surface water extent or were recently developed to address issues with more common methods. Table 3 provides details of each index, including spectral bands, equation, and primary source. 

In addition to the surface water indices, several methods of water detection have also been developed specifically for semi-arid regions. These include the simple water index (SWI) [21], a simple decision tree from Gond et al. [15] that uses the normalized difference vegetation index (NDVI), the normalized difference water index (NDWI), and the first shortwave infrared band (SWIR1), and a simple decision tree from Kaptue [14] that used NDVI and the modified normalized difference water index (MNDWI) (Table 3). 

Simple Water Index (SWI): The SWI method was developed by Malahlela [21] to avoid the problem of identifying optimal thresholds, and to maximize the differences between water and land cover types commonly confused with water such as green vegetation, shadows, and built-up areas. Non-water values are automatically nullified in the equation and the resulting values that are greater than five are classified as land cover types that have a similar response to water, while those values below five are classified as water. This method was derived specifically for an arid/semi-arid region in South Africa. The original study found that SWI had superior performance to the AWEI and MNDWI methods, however it is not widely used outside of the original paper. 

Gond Method: Gond and colleagues [15] developed a simple decision tree method using the VEGETATION instrument for identifying water bodies in the Sahel. This method was developed specifically to address the wide range of water body types and the variation in the surrounding landscape associated with seasonal changes in the region. First, the difference between NDVI and the normalized difference moisture index (NDMI) is calculated and the average is computed using a moving window of 45 pixels-squared. Next, the difference between this average and the original difference is calculated and pixels with a value greater than 0.08 are kept as potential water bodies. Then, a moving average of 45 pixels-squared is calculated for SWIR1 and the difference between the average and the original SWIR1 band is calculated. Pixels with a value of 0.05 or greater are kept as potential water bodies. Finally, the two outputs are combined using an “AND” function, and any pixels that satisfy both are classified as water. One potential source of error identified in the original paper is confusion of clouds for water; the authors suggest using a separate cloud mask to address this issue. Additionally, they suggest that this method may not work on water bodies that are large enough to impact the regional average of the moving window average. The Gond [15] simple decision tree does not perform well in areas with dense or moist vegetation because the contrast with water is diminished. This method was developed explicitly for dryland surface water detection. 

Sahel Water Body Product (SWBP): Kaptue and colleagues [14] developed a simple decision tree method for identifying water across the entire Sahel using 250-m MODIS imagery. The authors aimed to create a fast, efficient, and automatic means of identifying surface water. NDVI was used to distinguish waterbodies from dry soil and vegetation, however, since it may not distinguish water from snow, clouds, and bare land [3], MNDWI is also included to suppress bare land and built up areas. Pixels that had an NDVI value less than zero and an MNDWI value greater than zero are classified as water. 

Calibrating spectral indices: The primary methods of determining optimal thresholds of spectral indices include: the logical, programmatic threshold initially suggested in the literature proposing the index [5,6,7]; the eyeball approach, which relies on a subjective visual inspection of the classification [13,33]; the static optimal threshold method, which relies on in situ/reference calibration data and statistics [10,23]; and the dynamic threshold method which relies on image statistics and histograms [25,34]. In this study, for algorithms requiring a threshold, a Receiving Operator Characteristic (ROC) curve was generated using the pROC package in R. The ROC curve illustrates how different optimal threshold impact the sensitivity and specificity of the classification. It also allows for the calculation of the Area Under the Curve (AUC) which is an indicator of the overall utility of a given method as a classifier. The first set of random points was used for this calibration step. The optimal threshold was defined as the index value that minimized the specificity (true negative rate) and sensitivity (true positive rate), with equal weighting of each [35,36]. In other cases, differential weighting of different types of errors in the optimal threshold selection process may be appropriate depending on the purpose and goals of the analysis. The sensitivity and specificity were compared for each threshold using the following equation applied in R:

(1) $$D={\left({\left(1-Se\right)}^{2}+{\left(1-Sp\right)}^{2}\right)}^{\frac{1}{2}},$$

where D is the distance to (0,1), Se is sensitivity or true positive rate (TP = true positives/positives) and Sp is the specificity or true negative rate (TN = true negatives/negatives). The optimal threshold was selected as the index value with the lowest value for D. This calibration step was not applied for the three simple decision tree methods.

**Figure 3:**
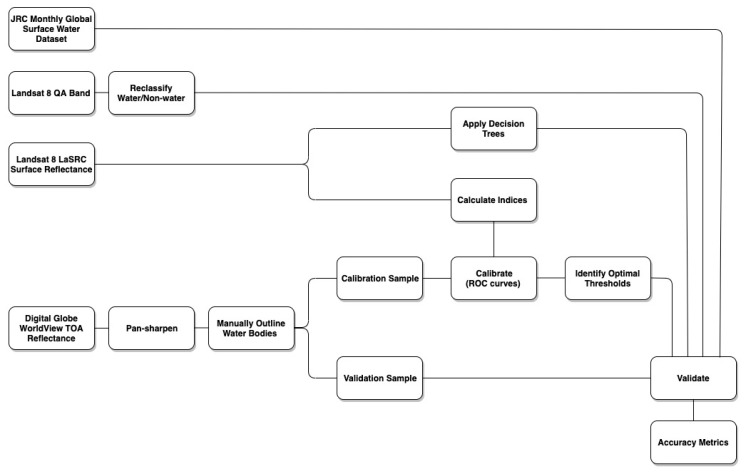
Flowchart of methods. Input datasets include the Joint Research Center (JRC) monthly global surface water dataset, Landsat 8 surface reflectance and QA band, and DigitalGlobe WorldView imagery.

Validating and comparing methods: The second set of random points was used to calculate the overall accuracy (OA) and the user’s and producer’s accuracy of all data after each image had been classified using the OT derived above. OA was calculated in R using the following equation:

(2) $$OA=\frac{TP+TN}{TSS},$$

where TP is the number of true positives, TN is the number of true negatives, and TSS is the total sample size. The user’s accuracy (UA) presents the accuracy from the user’s perspective and is also called the false positive rate, which indicates how many points were classified as water when they should have been classified as non-water. The producer’s accuracy provides the accuracy from the producer’s perspective and is also called the false negative rate, which indicates how many points were classified as non-water when they should have been classified as water. Figure 3 provides a flowchart of the methods and data used in this analysis. 

## 3. Results

### 3.1. Results of Existing Global Surface Water Dataset Assessment

**Figure 4:**
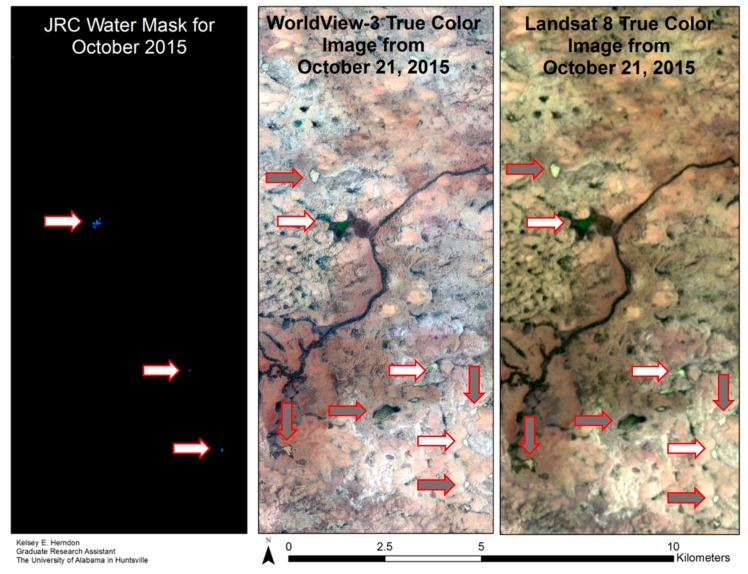
Sample results of JRC GSW dataset comparison. White arrows indicate water bodies identified in the JRC dataset. Grey arrows indicate water bodies not identified in the JRC dataset.

Both the JRC GSW and Landsat QA datasets practically eliminate false positive (FP) water identifications (Table 4). However, in doing so they underestimate the extent of surface water for the study area. For October 2015, the JRC GSW dataset had an OA of 0.84, with an FP rate of less than one percent but a false negative (FN) rate of 0.15. The Landsat QA water masks demonstrated similar results to the JRC GSW dataset, with OA ranging from 0.77 to 0.93, FP rate ranging from 0 to less than one percent, and the FN rate ranging from 0.02 to 0.22. The FN points for both global surface water datasets primarily consisted of water bodies that were small and turbid, as identified visually in the DG higher resolution imagery. The spectral signature of these water bodies can appear similar to certain soils and exposed rock, which may lead to misclassification if they are not included in the training dataset. Figure 4 illustrates underestimation of surface water extent provided by the JRC monthly surface water dataset compared to the reference dataset for the same month. 

### 3.2. Results of Spectral Index Assessment

Of the 15 spectral indices and bands tested, nine indices (SWIR1, SWIR2, NDMI, MNDWI/NDPI, WRI, TCW, AWEIsh, AWEInsh) had the best performance, with an OA across all scenes greater than 0.95 (Table 5). While all indices demonstrated some variability in the optimal threshold, six indices had optimal thresholds that varied less than 10% of the possible range of values for the given index. These included the NDMI, TCW, AWEIsh, NIR/R Ratio, NDWI, and NDVI. There were three indices that demonstrated both a high OA and a stable threshold: NDMI, TCW, and AWEIsh. The application of a single optimal threshold for these three indices would be appropriate for classifying water across space and time in the Sahel. NDWI, one of the most commonly used methods of surface water detection had relatively poor performance, with an OA of 0.88. NDWI was able to distinguish water from surrounding vegetation, but false positives were mainly due to confusion between water and bright, exposed bedrock. Additionally, NDWI failed to detect many smaller, more turbid water bodies.

### 3.3. Results of Decision Tree Assessment

The three simple decision trees created specifically for semi-arid regions had varied performance. Gond’s method using NDWI, NDVI, and SWIR1 had the best performance with an OA of 0.96. Kaptue’s method using MNDWI and NDVI had relatively poor performance with an OA of 0.88 and Malhala’s SWI had the worst performance with an OA of 0.83. 

## 4. Discussion

Three indices performed extremely well and had high threshold stability, making them excellent indices to apply for surface water detection in the Sahel: NDMI, TCW, and AWEIsh. While other indices exhibited high accuracies for individual scenes (SWIR1, SWIR2, NDPI/MNDWI, and WRI) they did not demonstrate optimal threshold stability. A stable optimal threshold indicates that a single threshold value is appropriate to use over multiple scenes across space and time, rather than having to generate a new threshold for each scene in order to maintain the highest accuracy. Optimal thresholds and OA varied greatly across time and space for other indices, suggesting that the static, simple thresholding of spectral indices may not be sufficient for mapping the variety of water body types in the Sahel. Additionally, simple decision trees developed specifically for the Sahel did not perform as well as these spectral indices. 

The worst performing indices were WI2015, with an OA of 0.69 and the NIR band with an OA of 0.75. WI2015, band ratios, and the NIR band also had the most unstable optimal threshold from scene to scene, indicating that these indices are not appropriate for monitoring surface water extent over space and time with a single threshold. These indices would require a new optimal threshold be generated for each Landsat scene in order to maintain the highest accuracy.

AWEIsh and AWEInsh were developed in part to solve the optimal threshold problem [10]. Previous work [10] demonstrated these two indices had high threshold stability across time and space, removing the need to identify an optimal threshold for each new scene. However, in this study, only AWEIsh demonstrated threshold stability, while optimal thresholds for AWEInsh varied greatly. 

One of the most widely used water detection indices, NDWI, is known to confuse built up areas for water bodies and such was the case for this study [7]. Other studies comparing commonly used methods of surface water detection in the Sahel and other semi-arid regions also found poor performance of the commonly used NDWI [23], likely due to the spectral similarity between built up areas, a known limitation of NDWI and certain landcover types in the region such as sand and exposed bedrock. MNDWI, another commonly used method of surface water detection, consistently confused vegetated areas with water bodies. This is a known issue related to the inability of MNDWI to distinguish between vegetation and inundated vegetation or vegetated water [14].

In many cases, differences in accuracy between the indices are very small, and may not be significant at the resource management level. The practical implications of differences in accuracies are an understudied topic that future work could address. 

Due to limitations in the overlap of high-resolution DG data and Landsat scenes, a non-probabilistic sample was used, whereby the chance of any given point within the Tahoua Region being selected was not equal across all points. Additionally, the sample datasets included mixed pixels, which may have decreased the accuracy of the algorithms. Studies that have separated mixed pixel from pure pixel classifications report an increase in accuracy once mixed pixels are removed from the study area [12]. The coefficients to calculate TCW were derived specifically for TOA reflectance and not surface reflectance, however, this index has been applied to surface reflectance products to identify surface water with some success [12]. Additionally, the method of deriving the reference dataset could have introduced errors as well. Future work could test for reproducibility in order to quantify the amount of error that might be introduced by using high-resolution imagery as a proxy for in-situ data. 

Finally, the JRC GSW dataset for the study area for October 2015 contained patterns of No Data that may have been due to the Landsat-7 SLC failure, however documentation for this dataset does not note this as a potential source of error. These areas of No Data may have contributed to the underestimation of surface water, however a visual inspection of areas with data present suggests there are still many areas of surface water in the reference dataset that are not present in the JRC GSW dataset. Future studies should expand the study area or time period to allow for additional overlap between the JRC GSW dataset and the high-resolution reference data. 

## 5. Conclusions

The results from this analysis demonstrate that existing global surface water datasets are not sufficient to address the limited availability of surface water in the Nigerien Sahel. The JRC GSW and Landsat QA datasets greatly underestimate the surface water extent for the study area, likely due to insufficient classification schemes for the Sahel or a training dataset that does not account for the spectral complexity of smaller, ephemeral water bodies in the Sahel. Algorithms calibrated specifically for the study area demonstrate a marked improvement in performance over these global datasets. Three spectral indices exhibited high accuracy and stable thresholds: NDMI, TCW, AWEIsh, indicating their appropriateness for monitoring changes in the extent of surface water in the Nigerien Sahel. NDWI, a commonly used index, had relatively poor performance and low threshold stability, along with WI2015, band ratios, and the NIR band. 

This paper provides a comprehensive comparison of fifteen spectral indices and three decision trees used to identify surface water from remote sensing data. An assessment of two global surface water datasets was also presented. Future work should expand this comparison in the Sahel beyond the Tahoua Region of Niger and could incorporate additional datasets, such as Sentinel-2 and other sources of high-resolution optical data, such as Planet. While spectral indices are the simplest and most commonly used methods of mapping surface water, analyses using machine learning approaches have also demonstrated their ability to more accurately map surface water over space and time in other regions [20]. These approaches should be included in future comparisons of water detection strategies in the Sahel. 

## Figures and Tables

**Table 1:** Landsat and DG imagery used.

Satellite/Sensor	Data Source	Image Resolution	Image ID	Date
Landsat 8 OLI	USGS ESPA	30 m	LC81910492015294LGN01	21 October 2015
			LC81910502017364LGN01	29 December 2017
			LC81910492017268LGN01	24 September 2017
			LC81910492016121LGN01	30 April 2016
WorldView-3	Digital Globe	2 m (pansharpened to 0.5 m)	1040010012220E00	21 October 2015
			10400100356E6500	29 December 2017
WorldView-2	Digital Globe	2 m (pansharpened to 0.5 m)	1030010070CF0800	24 September 2017
			10300100547AA300	30 April 2016

**Table 2:** In-situ surface water metrics (dates of the images are provided in Table 1).

DG Scene	Scene Area (km^2^)	Water Surface Area (km^2^)	Water Perimeter (km)	Rasterized Surface Area (km^2^)
1040010012220E00	1463.17	2.08	115.4	1.95
10400100356E6500	1576.51	2.26	58.92	2.23
1030010070CF0800	1923.60	8.78	181.26	8.54
10300100547AA300	1988.15	4.77	19.23	4.77

**Table 3:** Methods of surface water detection.

Name	Citation	Equation	Bands Used					
			B	G	R	NIR	SWIR1	SWIR2
Near Infrared	-	NIR	-	-	-	X	-	-
SWIR 1	-	SWIR 1	-	-	-	-	X	-
SWIR 2	-	SWIR 2	-	-	-	-	-	X
NIR-Red Ratio	13	NIR/RED	-	-	X	X	-	-
Red-Green Ratio	13	RED/GREEN	-	X	X	-	-	-
Normalized Difference Water Index (NDWI)	5	NDWI = (GREEN − NIR)/(GREEN + NIR)	-	X	-	X	-	-
Normalized Difference Moisture Index (NDMI)	6	NDMI = (NIR − SWIR)/(NIR + SWIR)	-	-	-	X	X	-
Modified Normalized Difference Water Index (MNDWI)	7	MNDWI = (GREEN − SWIR)/(GREEN + SWIR)	-	X	-	-	X	-
Normalized Difference Pond Index (NDPI)	13	NDPI = (SWIR − GREEN)/(SWIR + GREEN)	-	X	-	-	X	-
Water Ratio Index (WRI)	8	WRI = (GREEN + RED)/(NIR+ SWIR)	-	X	X	X	X	-
Tasseled Cap Wetness (TCW)	9	TCW = 0.1511 × BLUE + 0.1973 × GREEN + 0.3283 × RED +0.3407 × NIR − 0.7117 × SWIR1 − 0.4559 × SWIR2	X	X	X	X	X	X
Automated Water Extraction Index (AWEIsh)	10	AWEI(sh) = BLUE + 2.5 × GREEN − 1.5 × (NIR+SWIR1) − 0.25 × SWIR2	X	X	-	X	X	X
Automated Water Extraction Index (AWEInsh)	10	AWEI(nsh) = 4 × (GREEN − SWIR1) − (0.25 × NIR + 2.75 × SWIR1)	-	X	-	X	X	-
Normalized Difference Vegetation Index (NDVI)	11	NDVI = (NIR − RED)/(NIR + RED	-	-	X	X	-	-
WI2015	12	1.7204 + 171(GREEN) + 3(RED) + 70(NIR) + 45(SWIR1) + 71(SWIR2)	-	X	X	X	X	X
MNDWI and NDVI	14	Water where NDVI < 0 and MNDWI > 0	-	X	X	X	X	-
Simple Water Index (SWI)	21	SWI = 1/$\sqrt{\left(BLUE-SWIR1\right)}$, values <5 are classified as water	X	-	-	-	X	-
NDWI, NDVI, SWIR1	15	(1) NDVI − NDWI; (2) Average moving window of (1) (3) (1) − (2) (4) (3) > 0.8 kept as potential water (5) Average moving window of SWIR1 (6) (5) − SWIR1 (7) (6) > −0.1 kept as water	-	X	X	X	X	-

**Table 4:** Accuracy results for global datasets.

Dataset	Date	OA	FP	FN
Landsat 8 QA band	21 October 2015	0.82	<1%	0.18
	29 December 2017	0.78	0	0.22
	24 September 2017	0.93	0	0.07
	30 April 2016	0.77	0	0.20
JRC GSW	October 2015	0.84	<1%	0.15

**Table 5:** Accuracy results for indices.

Algorithm	Scene 1 (Calibrated to Scene 1)		Scene 2 (Calibrated to Scene 2)		Scene 3 (Calibrated to Scene 3)		Scene 4 (Calibrated to Scene 4)		All Scenes (Calibrated to All Scenes)	
	Optimal Threshold	OA	Optimal Threshold	OA	Optimal Threshold	OA	Optimal Threshold	OA	Optimal Threshold	OA
NIR	0.2965	0.70	0.4104	0.49	0.1659	0.89	0.2193	0.79	0.2338	0.75
SWIR 1	0.3032	0.96	0.3450	0.98	0.2110	0.99	0.2750	0.99	0.2522	0.97
SWIR 2	0.2035	0.95	0.2440	0.98	0.1210	0.99	0.2090	0.99	0.1652	0.97
NIR/R Ratio	1.2857	0.73	1.1587	0.96	1.1205	0.95	1.1754	0.63	1.1859	0.82
R/G Ratio	1.3935	0.84	1.4621	0.83	1.2413	0.94	1.2600	0.99	1.3774	0.84
NDWI	−0.2817	0.83	−0.2548	0.96	−0.2329	0.96	−0.1976	0.96	−0.2498	0.88
NDMI	−0.0365	0.96	0.0158	0.98	0.0149	0.98	−0.0753	0.99	0.0149	0.98
MNDWI	−0.3378	0.97	−0.2082	0.98	−0.3335	0.99	−0.3300	0.99	−0.3350	0.98
NDPI	0.3378	0.97	0.2080	0.98	0.3340	0.99	0.3300	0.99	0.3350	0.98
WRI	0.6056	0.97	0.7320	0.98	0.5799	0.97	0.6253	0.99	0.6250	0.97
TCW	−0.1047	0.96	−0.0555	0.98	−0.0803	0.99	−0.1410	0.99	−0.1118	0.98
AWEIsh	−0.4187	0.96	−0.4455	0.98	−0.3893	0.98	−0.4040	0.99	−0.4078	0.98
AWEInsh	−1.4701	0.96	−1.4995	0.98	−1.0600	0.99	−1.4200	0.99	−1.3835	0.98
SWI	-*	0.81	-*	0.95	-*	0.80	-*	0.77	-*	0.83
NDVI	0.1250	0.73	0.0735	0.96	0.0568	0.95	0.0806	0.63	0.0850	0.82
WI2015	80.0887	0.79	98.5396	0.90	52.8095	0.94	63.4000	0.97	66.6648	0.69
Kaptue	-*	0.87	-*	0.91	-*	0.95	-*	0.77	-*	0.88
Gond	-*	0.93	-*	0.97	-*	0.95	-*	0.97	-*	0.96

* optimal thresholds not calculated for simple decision tree methods.
